# Prediction of gross extrathyroidal extension in papillary thyroid carcinoma using a deep learning radiomics model based on post-processing techniques for multi-phase contrast-enhanced MRI images

**DOI:** 10.3389/fendo.2026.1917885

**Published:** 2026-09-29

**Authors:** Yun Zeng, Xinyi Li, Lang Tang, Xiaoli Xie, Anwei Mao, Bin Song

**Affiliations:** 1 Department of Radiology, Minhang Hospital, Fudan University, Shanghai, China; 2 Department of Ultrasound, Minhang Hospital, Fudan University, Shanghai, China; 3 Department of Pathology, Minhang Hospital, Fudan University, Shanghai, China; 4 Department of General Surgery, Minhang Hospital, Fudan University, Shanghai, China

**Keywords:** deep learning, extrathyroidal extension, papillary thyroid carcinoma, radiomics, spatial features

## Abstract

**Background:**

Gross extrathyroidal extension (ETE) affects surgical management and prognosis in papillary thyroid carcinoma (PTC), yet noninvasive assessment is difficult.

**Methods:**

In this retrospective exploratory feasibility study, 101 patients with pathologically confirmed PTC who underwent multi-phase contrast-enhanced MRI (MCE MRI) were analyzed. From the second and seventh contrast phases, four derived image types (ADD, ADD/SUB, Max, Min) were generated. Deep features from six image modalities were extracted using fine-tuned ResNet101, and radiomics classifiers were trained to predict gross ETE. Logistic regression identified independent clinical and MRI predictors, which, together with the deep learning radiomic signatures (DLR_signature) derived from the best-performing derived image type and algorithm, were integrated into a nomogram. Model performance was assessed by ROC, calibration, decision curve analysis, and ten-fold cross-validation.

**Results:**

Gross ETE was present in 37/101 cases (36.6%). The Min-modality deep transfer learning model achieved AUCs of 0.852 (training) and 0.853 (validation); the corresponding radiomics classifier had AUCs of 0.870 and 0.805. Multivariate analysis identified mean apparent diffusion coefficient (ADC_mean) (P = 0.001) and protrusion_value (P < 0.001) as independent predictors. The integrated nomogram combining Min-modality DLR_signature, protrusion_value, and ADC_mean achieved AUCs of 0.956 (training) and 0.895 (validation), with average AUCs of 0.875 and 0.846 in cross-validation. Calibration and decision-curve analyses showed favorable performance in the current dataset.

**Conclusion:**

The proposed nomogram integrating a Min-modality DLR_signature with quantitative MRI parameters showed promising potential for the noninvasive prediction of gross ETE in PTC. These findings support the feasibility of using MCE MRI post-processing for preoperative ETE prediction.

## Introduction

Papillary thyroid carcinoma (PTC) accounts for approximately 84% of all thyroid cancers, and its incidence continues to climb (1, 2). Although most PTCs carry a good prognosis — with a 5-year survival rate of 98.5% — certain histological variants behave aggressively and recur more frequently (3, 4). Extrathyroidal extension (ETE), defined by tumor invasion beyond the thyroid capsule into adjacent tissues, is as a powerful predictor of recurrence and poor outcomes (5, 6). The eighth edition of the American Joint Committee on Cancer (AJCC) staging system recognizes two grades of ETE: microscopic ETE, which no longer upstages tumor, and gross ETE, which is designated as T3b when invasion is confined to strap muscles, and assigned higher T stages when deeper invasion involves structures such as the trachea, larynx, esophagus, or recurrent laryngeal nerve (7, 8). Several studies have shown that microscopic ETE does not significantly impact survival or recurrence, while gross ETE correlates with markedly worse prognoses and mandates more extensive surgical resection (9–12). These insights highlight the imperative to identify gross ETE preoperatively to avoid both undertreatment and overtreatment.

Neck ultrasound remains the frontline tool for preoperative thyroid cancer staging, excelling in the detection of lymph node metastasis and superficial gross ETE (13, 14). However, magnetic resonance imaging (MRI) consistently outperforms ultrasound in sensitivity and specificity for deep-structure invasion, rendering it superior for comprehensive assessment of gross ETE (15–17). Parallel advances in artificial intelligence have demonstrated that deep learning and radiomics analyses can extract subvisual phenotypes that predict tumor behavior within organs (18–20). However, no study has yet integrated these techniques with MRI to predict gross ETE in PTC. In other malignancies, delta-projection imaging of dynamic contrast studies and delta-radiomics models have quantified perfusion changes to estimate proliferative indices and invasive potential (21, 22), and enhanced CT–based delta-radiomics has successfully forecast lymphatic spread in thyroid cancer (23).

Building on recent developments, our study evaluates the predictive value of multi-phase contrast-enhanced (MCE) MRI with inter-phase image processing, integrating deep learning radiomics (DLR) and conventional MRI parameters, for assessing gross ETE in PTC.

## Methods

### Patients and study design

The requirement for informed consent was waived due to the retrospective nature of the study. The work has been reported in line with the STROCSS criteria (24). The medical records of 181 patients with pathologically confirmed PTC and available preoperative MRI examinations were initially screened. Patients were eligible if they had undergone thyroid MRI within 14 days before surgery and had postoperative histopathological confirmation of PTC. Of the 181 patients screened, 80 were excluded: 39 because the required multiphase contrast-enhanced MRI sequence was unavailable, 3 because of inadequate image quality, 3 because of incomplete pathological data, and 35 because the target lesion measured less than 10 mm. Consequently, 101 patients were included in the final analysis. In patients with multifocal PTC, 43 additional smaller lesions were excluded from the lesion-level analysis; these exclusions did not reduce the number of included patients. The detailed patient-selection process is presented in **Figure 1**.

**Figure 1:**
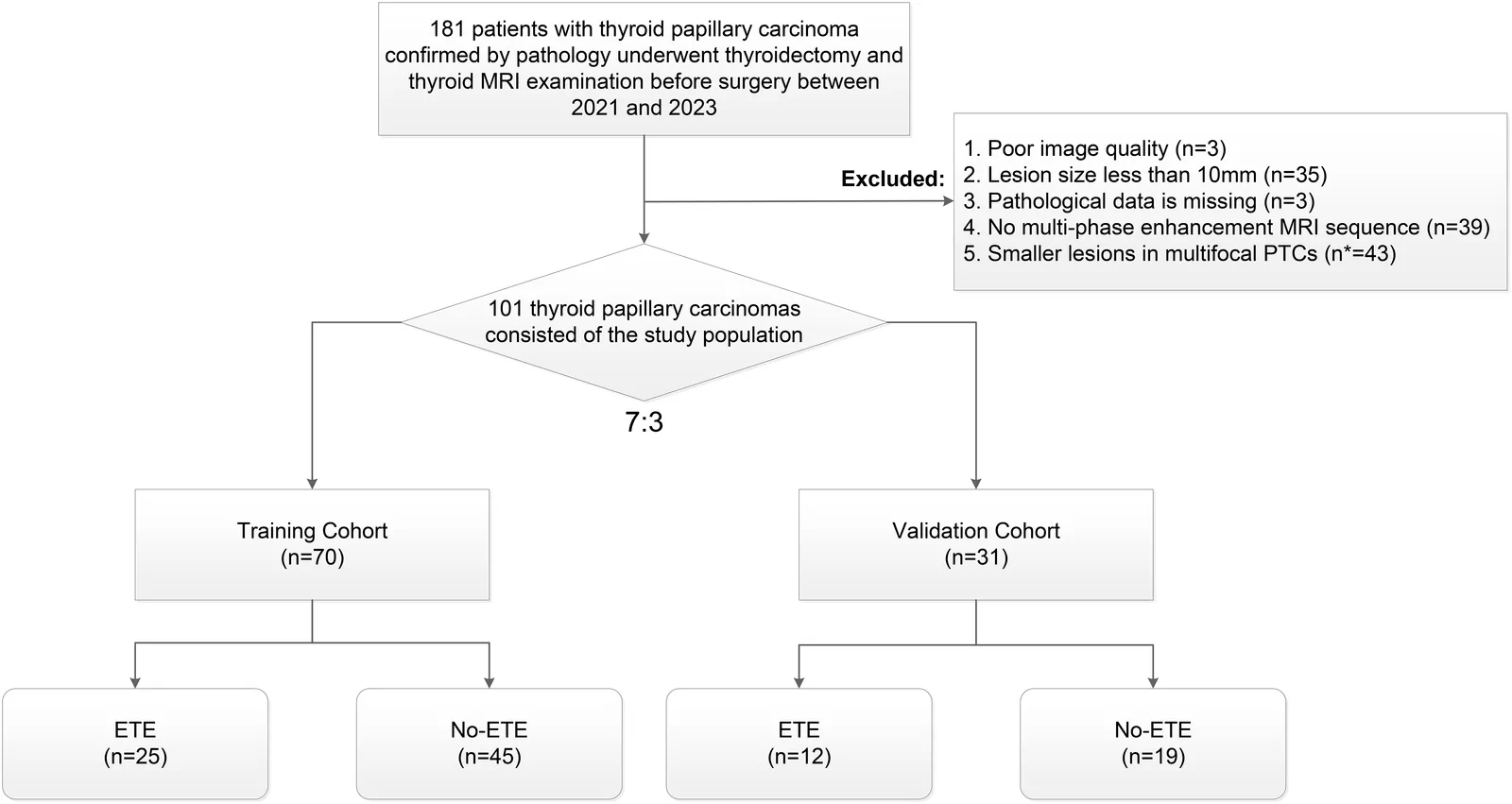
Flowchart of patient selection and cohort allocation. A total of 181 patients with pathologically confirmed papillary thyroid carcinoma (PTC) and available preoperative MRI examinations were screened. Eighty patients were excluded because of the absence of the required multiphase contrast-enhanced MRI sequence (n=39), inadequate image quality (n=3), incomplete pathological data (n=3), or a target lesion measuring less than 10 mm (n=35). The final cohort comprised 101 patients. In patients with multifocal PTC, 43 additional smaller lesions were excluded from the lesion-level analysis and were not counted as patient-level exclusions. n, patients; n*, lesions.

A total of 101 PTC lesions from 101 patients (30 males and 71 females; mean age, 45.3 ± 14.9 years; range, 12–77 years) met eligibility criteria. The lesions were randomly allocated to a training cohort (n=70) or a validation cohort (n=31) in a 7:3 ratio. Lesions were categorized into gross ETE and non-gross ETE groups. Gross ETE was defined according to the AJCC 8th edition as macroscopically evident tumor invasion into the strap muscles (T3b) or adjacent structures (T4). The final gross ETE status was retrospectively and independently reassessed by a senior pathologist based on a combined review of the operative records and corresponding histopathological slides. The operative records were used to determine whether macroscopic invasion had been observed during surgery and to identify the anatomical structures involved, whereas histopathological examination was used to confirm PTC and assess tumor extension into the sampled extrathyroidal tissues or resected adjacent structures. The reviewing pathologist was blinded to all preoperative imaging findings. The non-gross ETE group served as the negative class, encompassing both the absence of ETE and microscopic (minimal) ETE. All relevant clinical and imaging data were sourced from the medical records.

This study was conducted in accordance with the Declaration of Helsinki and approved by the Institutional Review Board. This retrospective study was designed as an exploratory feasibility study to investigate whether post-processing image transformation of MCE MRI could provide additional information for the preoperative prediction of gross ETE in PTC, and to develop a preliminary DLR-based nomogram.

### MRI acquisition

All MRI examinations were performed using a 1.5 Tesla system (GE Signa Excite HD; GE Healthcare, Milwaukee, WI, USA) equipped with a customized 8-channel neck coil (Chenguang Medical Technology Ltd). The MRI protocol comprised coronal and axial T2-weighted imaging (T2WI), axial T1-weighted imaging (T1WI), diffusion-weighted imaging (DWI; b values of 0 and 800 s/mm²), and axial multiphasic contrast-enhanced T1WI. A gadolinium-based contrast agent (Magnevist; Bayer Healthcare, Berlin, Germany) was administered intravenously at a rate of 3 mL/s (0.2 mL/kg), followed by a 20 mL flush of physiological saline. The MCE-MRI protocol comprised a pre-contrast mask phase (Phase 1) followed by six post-contrast phases acquired at 30 s (Phase 2), 60 s, 120 s, 180 s, 240 s, and 300 s (Phase 7). All seven phases of MCE MRI were acquired using the same predefined breath-hold protocol, consistent acquisition parameters, and identical spatial geometric parameters. Participants were instructed to avoid swallowing during image acquisition. Detailed MRI protocols can be found in the **Supplementary Materials** (**Supplementary Table E1**).

### Clinical and quantitative MRI parameters

Two radiologists (Z.Y. with 3 years and S.B. with 12 years of experience in thyroid MRI diagnosis, respectively) independently reviewed all MRI images using a picture archiving and communication system (PACS) workstation, remaining blinded to the histopathological findings. The data analysis incorporated both clinical indicators and quantitative MRI features. Clinical data — including age, sex, tumor location, the presence of Hashimoto’s thyroiditis (HT), focality status, and serum biomarkers (lactate dehydrogenase [LDH], thyroid-stimulating hormone [TSH], triiodothyronine [T3], thyroxine [T4], free triiodothyronine [F_T3], and free thyroxine [F_T4]) — were collected. Quantitative MRI parameters were measured on the slice demonstrating the largest tumor cross-section and comprised maximal lesion diameter, mean apparent diffusion coefficient (ADC_mean), minimum ADC (ADC_min), and protrusion_value. The protrusion_value was defined as the shortest perpendicular distance from the outermost point of the tumor to the line connecting the points of contact with the thyroid capsule (**Supplementary Figure E1**). All measurements represent the average of the values recorded by both radiologists.

### Image pre-processing

Four distinct image processing modalities – ADD, ADD/SUB, Max, and Min – were generated through different fusion methods of the early arterial phase (MCE_P2, 30 s) and the delayed phase (MCE_P7, 300 s) images (**Supplementary Figure E2**). Before image processing, two radiologists reviewed the paired phases and the derived fusion images. No significant spatial misalignment or ghosting artifacts were detected in the derived “Min,” “Max,” “SUB,” or “ADD” images used for training. All post-processing image analyses were performed using a commercially available workstation (Advantage Workstation 4.7, GE Healthcare).

The images from MCE_P7, MCE_P2, ADD, ADD/SUB, Max, and Min were exported in the Digital Imaging and Communications in Medicine (DICOM) format, resampled to a voxel size of 0.1×0.1×1 mm^3^ to standardize the input for deep learning and radiomics analysis, and underwent N4 bias correction to correct intensity inhomogeneity. Due to the small size of PTC lesions in this cohort, a 2D segmentation approach was used. Small tumors often had marginal slices with few tumor voxels, making them prone to partial-volume effects with nearby tissues. Anisotropic image resolution also increased boundary uncertainty between slices. To enhance segmentation consistency and simplify the model for the small sample size, the axial slice with the largest tumor cross-section was chosen for analysis. A radiologist (five years of experience in thyroid MRI) blinded to the pathologic results, independently delineated the regions of interest (ROIs) using ITK-SNAP software (version 3.8.0, http://www.itksnap.org/). To ensure anatomical consistency across all input channels, the ROI was manually delineated on the MCE_P7 images, which offered the optimal tumor-to-background contrast for boundary definition. This defined ROI mask was then propagated to the spatially aligned images of the other five modalities (MCE_P2, ADD, ADD/SUB, Max, and Min). All ROIs were then reviewed and finalized by a senior radiologist with 12 years of experience. To assess inter-observer reproducibility, a junior radiologist (three years of experience in thyroid MRI) independently segmented a random subset of 40 lesions; intraclass correlation coefficients (ICCs) were computed for all texture features, and only features with ICC≥0.80 were retained for further analysis. The detailed image preprocessing procedures are described in the **Supplementary Material** under “Additional Details of Image Preprocessing”.

### Deep transfer learning and radiomic feature extraction and selection

We extracted quantitative radiomic features from the six imaging modalities — MCE_P7, MCE_P2, ADD, ADD/SUB, Max, and Min — using Python (version 3.7.12) in conjunction with the Pyradiomics toolkit (version 3.1.0). For each modality, we selected a single axial slice corresponding to the largest tumor cross-section to ensure consistency. Deep transfer learning signatures (DTL_signatures) were generated by fine-tuning a pre-trained ResNet101 model (PyTorch, version 1.11.0) on these representative images; the resulting output probabilities served as deep learning descriptors (**Supplementary Figure E3**). During training, we applied random cropping and horizontal flipping to enhance the input data and improve generalization. The training and optimization procedures for ResNet-101 are described in detail in the “Deep Transfer-Learning Model Optimization” section of the **Supplementary Materials**. We then concatenated the handcrafted radiomic features with the DTL_signatures in a late fusion framework. For each imaging modality, features showing significant between-group differences were first identified using the independent-samples t test or Mann–Whitney U test, as appropriate, with a significance threshold of P < 0.05. Pairwise Pearson correlation coefficients were calculated to identify and remove highly collinear features (r>0.90), retaining only one feature from each correlated pair. Finally, we applied the Least Absolute Shrinkage and Selection Operator (LASSO) with five-fold cross-validation to the fused feature set, selecting those variables with non-zero coefficients at the regularization parameter (λ) that minimized cross-validated error. All data-driven feature selection, deep network training, fine-tuning, and classifier fitting were conducted solely in the training cohort for each imaging modality.

### Deep learning radiomics and nomogram model construction

In the training cohort, we performed feature selection separately for the radiomics and DLR pipelines. For each of the six imaging modalities (MCE_P7, MCE_P2, ADD, ADD/SUB, Max, Min), we trained six classifier types — logistic regression (LR), support vector machine (SVM), random forest, ExtraTrees, Naive Bayes, and Light Gradient Boosting Machine (LightGBM) — using the fused feature set to predict gross ETE. Hyperparameter optimization was conducted using only the training cohort through grid search combined with five-fold cross-validation. The diagnostic performance of the radiomic models derived from the six image types, as well as the DTL models, was assessed in both the training and validation cohorts using the most effective algorithm. The imaging modality and classifier yielding the highest internal-validation AUC were selected for the final DLR framework. The predictive probability output (DLR_signature) generated by the DLR model was subsequently used to construct a nomogram for predicting gross ETE in PTC.

We then performed multivariable logistic regression analysis on all quantitative MRI parameters in the training cohort to identify independent predictors of gross ETE in PTC. The final nomogram combined the DLR_signature with these independent predictors using a logistic regression framework. We evaluated the nomogram’s discrimination by area under the curve (AUC) of receiver operating characteristic (ROC) analysis, its calibration by plotting predicted versus observed probabilities and computing the Hosmer–Lemeshow statistic, and its clinical usefulness by decision curve analysis. The workflow of the study is shown in **Figure 2**. Definitions of Terms Used in the Manuscript are provided in **Supplementary Table E5**.

**Figure 2:**
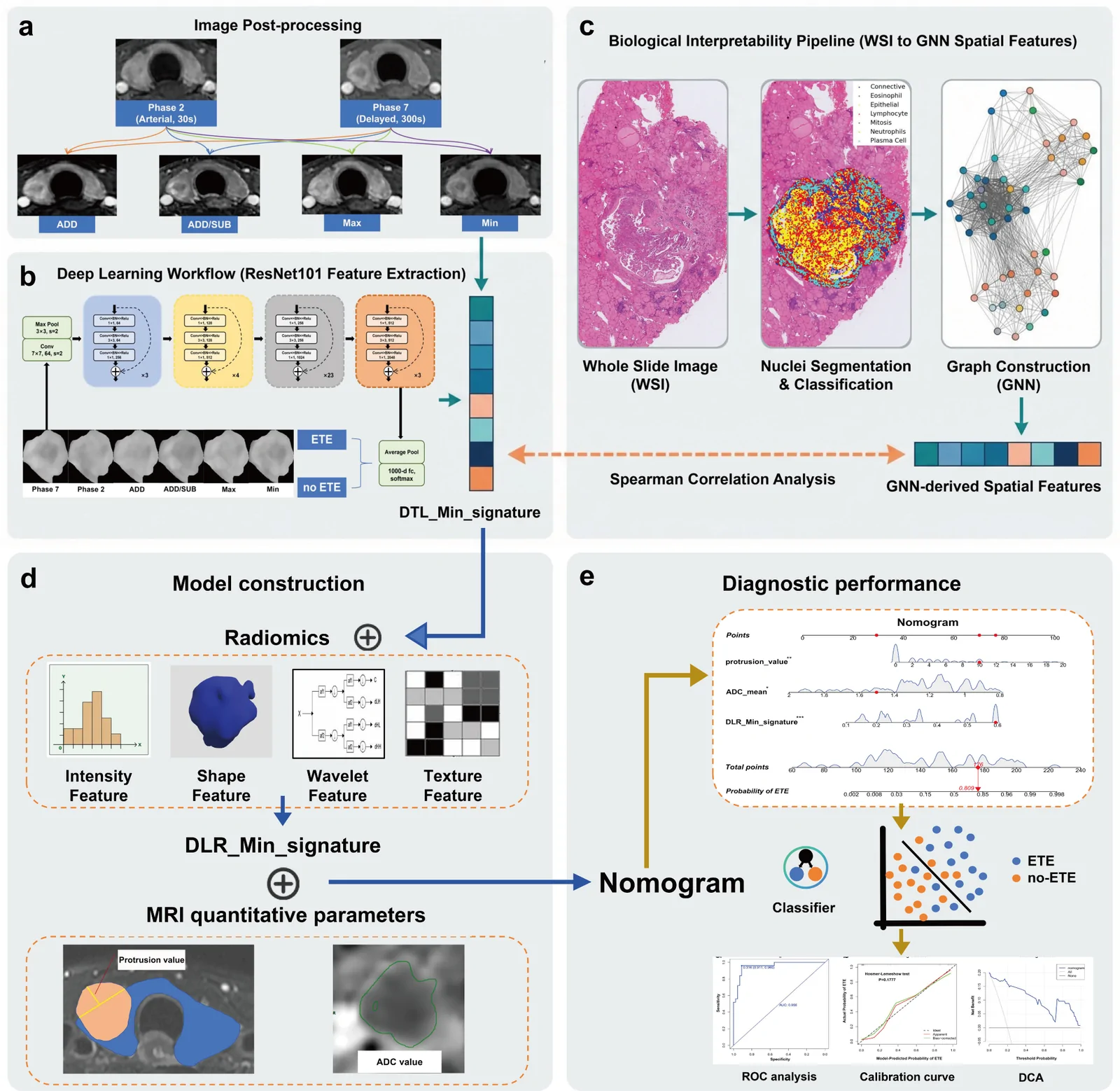
Diagram of the workflow for this study.

### Extraction of graph neural network-based spatial features and correlation analysis with the DTL_signature

Formalin-fixed paraffin-embedded (FFPE) tissue samples underwent hematoxylin and eosin (H&E) staining and were digitized by whole-slide scanning. Two-dimensional tumor regions were manually delineated in QuPath software on the resulting whole-slide images (WSIs). We extracted GNN–derived spatial features from the WSIs using the pipeline described in the **Supplementary Materials** (“WSI Processing and Spatial Feature Extraction”). To investigate the histopathological context of the DTL signature derived from the optimal derived image, we conducted a *post hoc* exploratory correlation analysis with GNN-derived spatial features. This aimed to generate hypotheses about potential imaging-pathology associations and was not intended as confirmatory validation.

### Statistical analysis

Data analysis was performed on the Onekey platform (version 4.9.1) using Python (version 3.7.12), with additional statistical analyses conducted using SPSS version 26.0 (IBM Corp., Armonk, NY, USA) and R software 9 (version 4.2.2, www.r-project.org). Continuous variables with a normal distribution were reported as mean ± standard deviation (SD), while non-normally distributed variables were reported as median and interquartile range (IQR). Categorical variables are expressed as percentages. The Shapiro-Wilk test assessed normality. We compared normally distributed variables with independent samples t-tests and non-normal distributed variables with the Mann-Whitney U test. Fisher’s exact test or chi-squared test evaluated differences in categorical data. Inter-observer agreement for quantitative parameters was evaluated using the ICC. Binary logistic regression analyzed clinical and MRI variables linked to gross ETE. Due to few gross ETE events, only select variables were included in the multivariable analysis, focusing on clinically relevant ones and those showing univariable associations. For clarity, ADC values were rescaled and examined per 0.1 × 10–^3^ mm^2^/s increment. We evaluated diagnostic model performance via ROC analysis, reporting AUC, accuracy, sensitivity, specificity, positive predictive value (PPV), and negative predictive value (NPV), and we performed internal validation by ten-fold cross-validation. Comparison of AUCs of different models was performed by DeLong test. To interpret the contributions of individual features in the DLR model, we applied Shapley Additive Explanation (SHAP) analysis. A nomogram was constructed using the “rms” package in R. Spearman correlation analysis was performed to evaluate the association between the DTL_signature and digital pathology spatial features. All statistical tests were two-tailed, and P < 0.05 was considered statistically significant.

## Results

### Clinicopathological characteristics and MRI features

The clinicopathological characteristics and MRI features of both the training and validation cohorts are shown in **Table 1**. No statistically significant differences were observed between the training and validation cohorts. Of the 101 lesions, 70 (69.3%) were assigned to the training cohort (mean age, 45 ± 14 years; 46 females and 24 males) and 31 (30.7%) to the validation cohort (mean age, 45 ± 17 years; 25 females and 6 males). Gross ETE was identified in 25 of 70 lesions (35.7%) in the training cohort and 12 of 31 (38.7%) in the validation cohort.

**Table 1:** The clinicopathological characteristics and MRI radiologic features for both training and validation cohorts.

Characteristic	Total	Training cohort	Validation cohort	P
	n=101	n=70	n=31	value
Age, years	45.34 ± 14.91	45.29 ± 13.92	45.45 ± 17.20	0.101
Sex, n (%)				0.132
Female	71 (70.3)	46 (65.7)	25 (80.6)	
Male	30 (29.7)	24 (34.3)	6 (19.4)	
HT, n (%)				0.602
Absent	72 (71.3)	51 (72.9)	21 (67.7)	
Present	29 (28.7)	19 (27.1)	10 (32.3)	
Tumor number, n (%)				0.871
Multifocal	25 (24.8)	17 (24.3)	8 (25.8)	
Unifocal	76 (75.2)	53 (75.7)	23 (74.2)	
Tumor size, mm	16.0 (13.0, 21.0)	17.0 (14.0, 22.5)	16.0 (12.0, 21.0)	0.349
Location, n (%)				0.892
Isthmus	4 (4)	4 (5.7)	0 (0)	
Left	39 (38.6)	26 (37.1)	13 (41.9)	
Right	58 (57.4)	40 (57.1)	18 (58.1)	
Protrusion_value, mm	3.00 (0.00, 6.00)	2.00 (0.00, 6.00)	3.00 (0.00, 6.00)	0.548
ADC_mean	1.17 (1.04, 1.33)	1.18 (1.02, 1.35)	1.17 (1.06, 1.28)	0.823
ADC_min	0.65 ± 0.26	0.65 ± 0.27	0.65 ± 0.25	0.496
LDH, U/L	143 (132, 157)	143 (131, 154)	143 (133, 157)	0.643
TSH, μIU/mL	2.71 (1.65, 3.47)	2.71 (1.64, 3.45)	2.71 (1.80, 3.56)	0.641
T3, nmol/L	1.67 (1.50, 1.75)	1.67 (1.52, 1.77)	1.67 (1.49, 1.69)	0.378
T4, nmol/L	98.2 (87.62, 100.5)	98.2 (87.8, 99.1)	98.2 (87.4, 101.5)	0.339
F_T4, pmol/L	16.5 (15.2, 17.3)	16.5 (15.3, 17.5)	16.5 (14.7, 17.1)	0.932
F_T3, pmol/L	4.79 (4.45, 4.85)	4.79 (4.52, 5.03)	4.76 (4.32, 4.79)	0.083
gross ETE, n (%)				0.774
Absent	64 (63.4)	45 (64.3)	19 (61.3)	
Present	37 (36.6)	25 (35.7)	12 (38.7)	

n, number of lesions; HT, Hashimoto’s thyroiditis; LDH, lactate dehydrogenase; TSH, thyroid stimulating hormone; T3, triiodothyronine; T4, thyroxine; F_T3, free triiodothyronine; F_T4, free thyroxine; ADC, apparent diffusion coefficient; ETE, extrathyroidal extension.

### Radiomics analysis

From the six image transforms (MCE_P7, MCE_P2, ADD, ADD/SUB, Max, and Min), we extracted a total of 4998 radiomic features, including 162 first-order features, 14 shape features, and a spectrum of second-order statistical features as follows: 198 gray level co-occurrence matrix (GLCM) features, 144 gray level run length matrix (GLRLM) features, 144 gray level size zone matrix (GLSZM) features, 45 neighboring gray tone difference matrix (NGTDM) features, and 126 gray level dependence matrix (GLDM) features. The interobserver ICCs ranged from 0.27 to 0.99. After reproducibility filtering (ICC≥0.80) and LASSO selection, each modality retained a parsimonious feature set (5 features for MCE_P7, 9 for MCE_P2, 12 for ADD, 8 for ADD/SUB, 3 for Max, and 8 for Min). When we applied optimal machine-learning classifiers to these reduced feature pools, the Min-based model using LightGBM outperformed all others in the validation cohort (**Supplementary Table E2**).

### Deep transfer learning models

**Supplementary Table E3** presents the diagnostic efficacy of the DTL model in predicting gross ETE across six image processing modalities. In the validation cohort, the Min-modality DTL model achieved an AUC of 0.853, substantially outperforming the other models: MCE_P7 (AUC 0.586), MCE_P2 (0.632), ADD (0.700), ADD/SUB (0.660), and Max (0.697). We therefore adopted the Min-modality DTL model output probability — hereafter the DTL_Min_signature — as the core input for our DLR framework. Gradient-weighted Class Activation Mapping (Grad-CAM) images of the DTL models for predicting gross ETE, were generated for the six different image processing modalities (**Supplementary Figure E4**). As shown in **Supplementary Figure E5**, the learning curves demonstrate the performance of the ResNet101 architecture within the Min image modality.

### Deep learning radiomics models

In the DLR framework, we adopted a late-fusion strategy to integrate the 13 LASSO-selected features (**Supplementary Table E4**) with the DTL_Min_signature. We then trained a LightGBM classifier using the combined features. This model (DLR_Min_signature) achieved the optimal diagnostic performance, yielding an AUC of 0.918 (95% CI: 0.852–0.984) in the training cohort and an AUC of 0.846 (95% CI: 0.681–1.000) in the validation cohort. **Supplementary Figure E6** displays two representative PTC cases on Min modality images, along with feature importance ranking and a SHAP plot. Although five of the 13 features made major contributions to the prediction, the DTL_Min_signature emerged as the single most influential predictor.

### Development and validation of the nomogram model

In the training cohort, univariate analysis revealed that several clinical and imaging variables differed significantly between tumors with and without gross ETE, but multivariate logistic regression distilled these to two independent predictors: protrusion value (odds ratio [OR] 1.47 per millimeter increase; P < 0.001; ICC value = 0.896) and mean ADC (OR 0.53 per 0.1×10^-3^ mm²/s increase; P = 0.001; ICC value = 0.841), as shown in **Table 2**. Thus, higher ADC_mean values were associated with lower odds of gross ETE. Incorporating these two predictors with the DLR_Min_signature into a single logistic regression model yielded a nomogram capable of estimating each patient’s individual probability of gross ETE, as illustrated in **Figure 3**. In the training cohort, this nomogram achieved an AUC of 0.956 (95% CI, 0.909–1.000), and an AUC of 0.895 (95% CI, 0.784–1.000) in the validation cohort, as illustrated in **Figure 4**. The calibration plots demonstrated excellent agreement between predicted and observed rates of ETE (Hosmer–Lemeshow P = 0.178 in training; P = 0.451 in validation). Decision curve analysis confirmed that the nomogram provided a positive net clinical benefit across nearly all threshold probabilities (0–100% in training; 5–100% in validation), as illustrated in **Figure 4**. Finally, 10-fold cross-validation yielded mean AUCs of 0.875 in the training cohort and 0.846 in the validation cohort (**Supplementary Figure E7**).

**Table 2:** Univariate and multivariate analysis to identify factors associated with gross ETE in training cohort.

Variables	Univariate analysis		Multivariate analysis			
	no-ETE_max (N = 45)	ETE_max (N = 25)	Odds ratio (95% CI)	P value	Odds ratio (95% CI)	P value
Protrusion_value (mm)	0	5	1.34	<0.001*	1.47	<0.001*
	(0.00, 3.00)	(3.00, 9.00)	(1.14, 1.59)		(1.21, 1.79)	
ADC_mean	1.28 (1.13, 1.37)	1.03 (0.96, 1.23)	0.03	0.005*	0.53 (0.36, 0.78)	0.001*
			(0.00, 0.42)			
ADC_min	0.70 ± 0.24	0.55 ± 0.29	0.11	0.019*		
			(0.02, 0.73)			
TSH, μIU/mL	2.59 (1.35, 2.92)	2.71	1.5	0.009*		
		(2.55, 3.99)	(1.07, 2.11)			

n, number of lesions; ETE, extrathyroidal extension; ADC, apparent diffusion coefficient; CI, confidence interval; TSH, thyroid stimulating hormone.

*, P < 0.05.

**Figure 3:**
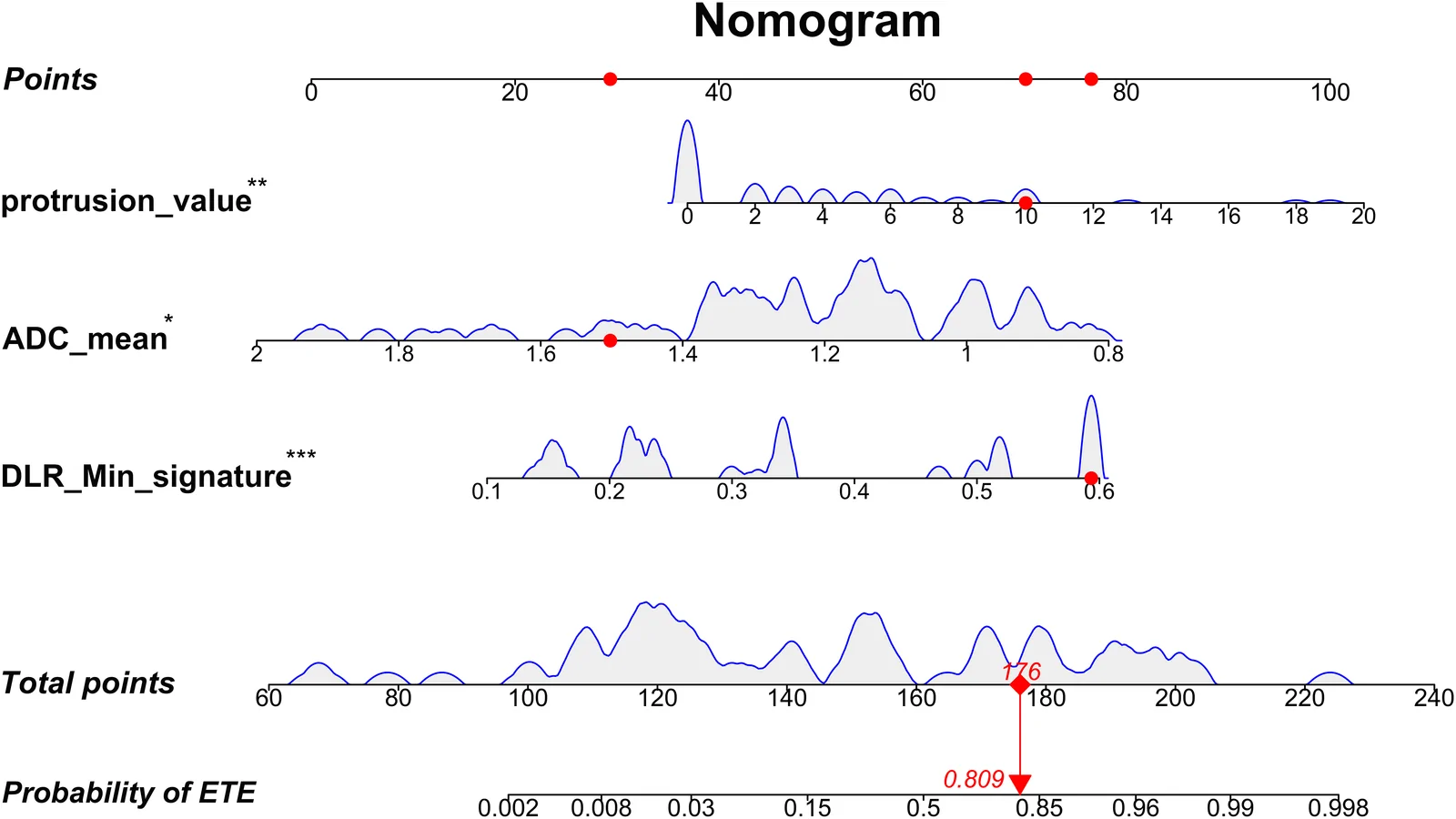
The nomogram for predicting gross ETE probability in PTC. Identify patient values on each variable axis, draw lines to the Points axis to find scores, add these scores on the Total Points axis, and project the total downward to find the gross ETE Probability. *, P < 0.05; **, P < 0.01; ***, P < 0.001.

**Figure 4:**
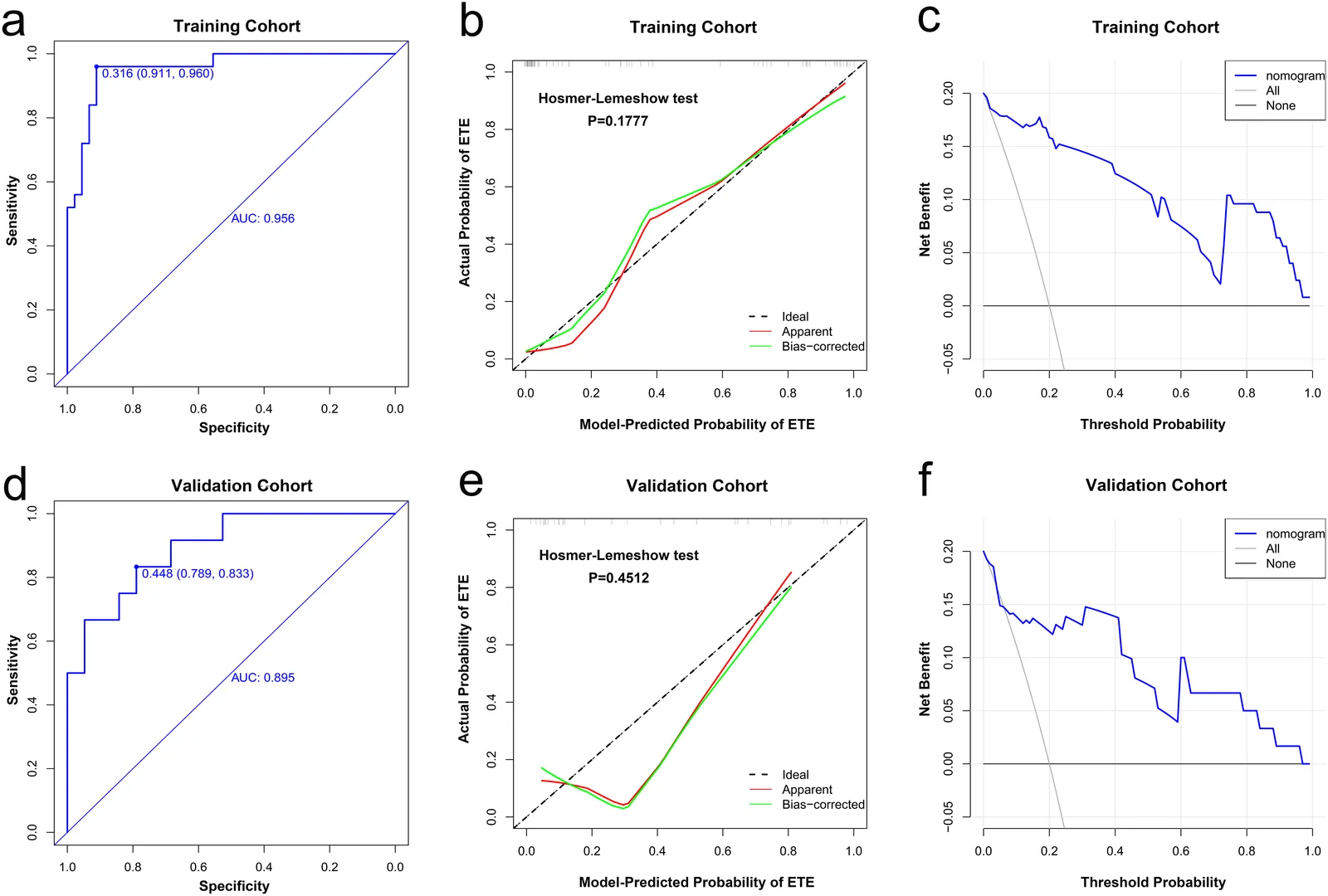
Validation of the nomogram for distinguishing gross ETE from no gross ETE in PTC showed: **(a)** a training cohort ROC curve with an AUC of 0.956, sensitivity of 96.0%, and specificity of 91.1% at a threshold of 0.316; **(b)** a Hosmer-Lemeshow test P-value of 0.1777 for calibration; **(c)** decision curve analysis; **(d)** a validation cohort ROC curve with an AUC of 0.895, sensitivity of 83.3%, and specificity of 78.9% at a threshold of 0.448; **(e)** a Hosmer-Lemeshow test P-value of 0.4512 for calibration; and **(f)** decision curve analysis.

ROC analyses compared a conventional MRI model, senior radiologist assessment, and nomogram model. The nomogram achieved the highest AUC of 0.935, outperforming both the conventional MRI model (AUC 0.852, P = 0.005) and radiologist assessment (AUC 0.664, P<0.001) (**Supplementary Figure E8**). The MRI model also significantly surpassed radiologist assessment (P<0.001).

### Correlation analysis between the DTL_Min_signature and GNN-derived spatial features

A total of 101 WSIs of PTC lesions were initially collected. We excluded 31 WSIs because of blurring, leaving 70 WSIs for analysis. We extracted 112 GNN-derived spatial features and retained 64 features after excluding 48 features with incomplete data.

In the deep learning radiomics model, five parameters contributed most to prediction performance, with DTL_Min_signature emerging as the most influential independent predictor. Correlation analysis showed a positive association (r > 0.3) between the DTL_Min_signature and four GNN-derived epithelial spatial features – Epithelial_Degree_StdDev, Epithelial_Degree_Mean, Epithelial_Connection_Frequency, and Epithelial_Eigenvector_StdDev. All of these features are associated with the spatial characteristics of epithelial cells (**Figure 5**).

**Figure 5:**
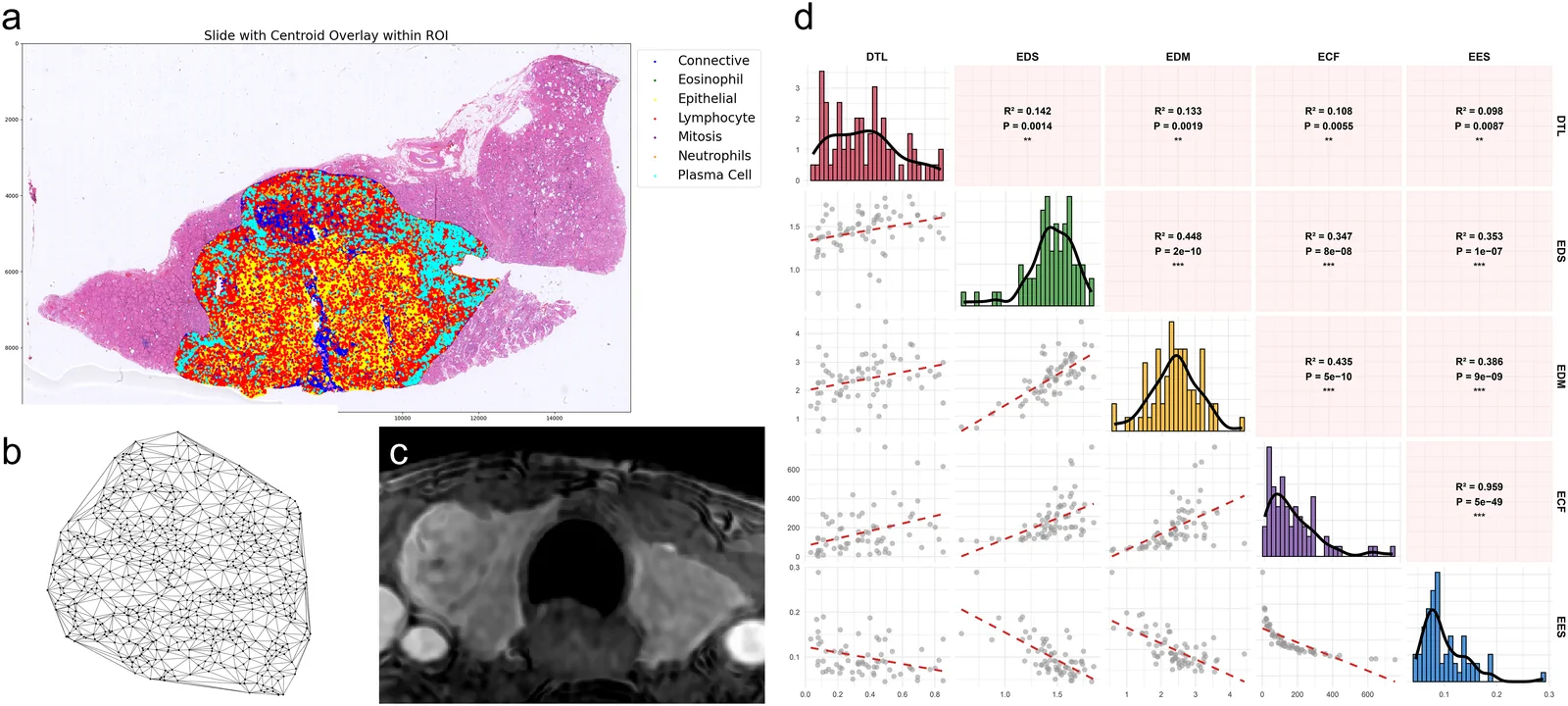
Visualization of representative features and correlation analysis. (a–c) Representative images from a 47-year-old female patient with right-sided papillary thyroid carcinoma (PTC) and no gross extrathyroidal extension (ETE). **(a)** Whole Slide Image (WSI) cell classification map. **(b)** Graph Neural Network (GNN) graph structure. **(c)** Visualization of the ‘min’ modality map. **(d)** Scatter plot matrix illustrating pairwise associations between the DTL_Min_signature and selected GNN spatial features. Diagonal panels display the distribution density of each variable. The upper triangle presents the coefficient of determination (R^2^) and P-values, while the lower triangle depicts scatter plots with fitted linear regression lines. Notably, four features demonstrated a correlation coefficient (r) > 0.3 with the DTL_Min_signature: Epithelial_Degree_StdDev, Epithelial_Degree_Mean, Epithelial_Connection_Frequency, and Epithelial_Eigenvector_StdDev. These metrics collectively reflect the spatial characteristics of epithelial cells.

## Discussion

In this exploratory feasibility study, the Min-modality DLR model exhibited superior internal validation performance compared to the six imaging modalities evaluated. The nomogram, which integrates the DLR_Min_signature with protrusion_value and ADC_mean, further demonstrated significant potential for discriminating preoperative gross ETE in PTC. These findings indicate that the integration of DLR_Min model outputs with traditional quantitative parameters may effectively capture imaging information pertinent to intratumoral heterogeneity and macroscopic invasion characteristics.

Gross ETE in PTC increases both mortality and recurrence risks, yet reliable preoperative detection remains elusive. An ultrasound-based deep-learning model recently achieved an AUC of 0.88 for predicting gross ETE (15). Most existing studies, however, focus on overall ETE: a meta-analysis of ultrasound-based radiomics reported pooled sensitivity of 77%, specificity of 84%, and an AUC of 0.82 for ETE detection (25). In CT domain, a conventional radiomics model achieved an AUC of 0.797 in the test cohort (26), while an iodine map-based radiomics approach achieved an AUC of 0.84 in the validation set (27). In comparison, our unimodal DLR model achieved an AUC of 0.846 in the validation cohort, demonstrating its robust diagnostic performance.

MRI-based radiomics models have demonstrated robust diagnostic performance in preoperatively predicting ETE in patients with PTC (28). However, conventional approaches typically analyze only single-phase data, leaving much of the rich temporal information in multi-phase contrast-enhanced (MCE) MRI untapped. Recent advances in image processing have begun to address this gap. Digital subtraction between sequential acquisitions can reveal subtle perfusion changes in brain imaging, thereby improving early diagnosis (29), and multimodal fusion aids neurosurgeons by integrating anatomical and functional data for precise skull-base tumor resections (30). Additionally, phase-specific subtraction on dynamic MRI accurately quantifies hepatocellular carcinoma necrosis following chemoembolization, with arterial-phase subtraction proving most reliable (31). These examples illustrate how tailored post-processing techniques can reveal physiological and pathological details that may be overlooked by static images alone.

Building on these innovations, we generated six distinct MCE MRI transforms and extracted both deep transfer learning signatures and handcrafted radiomic features from each. When we compared machine learning–based radiomics and deep transfer learning models across these modalities, both achieved their highest validation AUCs using Min modality images. One explanation is that minimum-value fusion might retain low-enhancing components across contrast phases, offering additional insights into intratumoral heterogeneity and the tumor–thyroid interface. However, this theory is speculative, and the Min modality’s effectiveness needs validation in an independent study. The observed diagnostic performance was encouraging compared with previously reported single-phase MRI or ultrasound-based approaches. It showed favorable calibration and decision-curve characteristics in the current dataset, supporting its potential as a sensitive, noninvasive tool for tailored surgical planning in PTC.

Deep learning radiomics synergistically combines the convolutional neural network-derived signatures with handcrafted radiomic descriptors to capture both high-level imaging patterns and quantitative texture phenotypes. However, the optimal strategies for feature integration concerning the presence or absence of gross ETE in PTC remain insufficiently explored. Recent research has demonstrated the promise of such fusion approaches for predicting cervical lymph node metastasis in PTC by integrating deep learning-derived features with radiomics metrics (19, 32). In the present study, we adapted this paradigm to gross ETE prediction by developing a logistic regression nomogram that integrates a post-fusion DLR_signature — extracted from Min modality MCE-MRI images — with key quantitative MRI parameters (protrusion_value and ADC_mean). This combined model exhibited excellent predictive performance.

Research has underscored the value of diffusion metrics in preoperative ETE assessment. Hao et al. (33) found significantly lower ADC percentiles in PTC patients with ETE, particularly at the 5th, 25th percentiles, and median, with the 5th percentile being most effective (AUC = 0.757). Hu et al. (34) similarly demonstrated that mean ADC values derived from DWI at b-values of 300, 500, and 800 s/mm² could distinguish PTCs with and without ETE, with b = 500 s/mm² yielding the highest accuracy. In our cohort, ADC_mean emerged as an independent risk factor for gross ETE, with an AUC of 0.721 in the training cohort, consistent with these prior findings. The lower ADC values observed in PTCs with gross ETE likely reflect increased cellular density and restricted molecule diffusion due to higher proliferative activity and enlarged nuclei. In addition to diffusion metrics, we found that protrusion value independently predicted gross ETE. Tumors exhibiting greater outward extension beyond the thyroid capsule — quantified by higher protrusion values — correspond to more aggressive growth patterns and a higher likelihood of capsular breach. By combining ADC_mean and protrusion value with Min-modality DLR_signature, the resulting nomogram incorporates both microstructural and macroscopic invasion features to optimize preoperative gross ETE prediction.

The exploratory analysis utilizing GNN for pathology identified correlations between the MRI-derived DTL_Min_signature and epithelial topology metrics, with a particular emphasis on Epithelial_Degree_StdDev. One possible interpretation is that macroscopic MRI enhancement patterns may covary with aspects of microscopic tumor architecture. However, this interpretation remains conjectural. Both the DTL signature and the GNN-derived features are high-dimensional computational constructs that cannot be directly linked to specific cellular processes or molecular pathways based solely on correlation. Consequently, the current findings should be regarded as hypothesis-generating rather than providing mechanistic evidence. Future research should consider integrating immunohistochemical, genomic, transcriptomic, or spatial-transcriptomic data to further elucidate whether the identified associations correspond to specific biological pathways associated with tumor invasion.

Several limitations warrant consideration. First, this retrospective, single-center feasibility study involved 101 patients, including 37 with gross ETE, resulting in a limited sample size that may affect model precision and increase overfitting risk. To address this, we used an ImageNet-pretrained ResNet101, applied dimensionality reduction and feature selection in the training cohort, limited the final model to 13 predictors, and performed ten-fold cross-validation. Despite these measures, the small sample size means the findings should be considered preliminary. Second, while the internal validation cohort wasn’t used for feature selection, deep network training, or classifier fitting, it served to compare imaging modalities and algorithms. This makes it an internal validation cohort, not a fully independent test cohort, which might have caused selection bias in the validation AUCs. Given the small cohort size and multiple evaluations, the perceived superiority of the Min modality and the performance of the final DLR model and nomogram need validation in an independent external test cohort. Third, the single-center design and lack of external testing restrict the findings’ generalizability across different institutions, populations, MRI scanners, and protocols. Future multicenter studies should predefine and lock the image-processing method, feature-selection, classifier, model parameters, and decision threshold before testing on an independent cohort. Thus, this study mainly demonstrates the feasibility of MCE MRI post-processing for gross ETE prediction, rather than providing definitive validation of a clinically deployable model. Fourth, only the largest tumor cross-section was analyzed, which minimized partial-volume effects and simplified segmentation, but may have missed the tumor’s full spatial heterogeneity and focal invasion beyond the chosen slice. No comparison with multi-slice or whole-volume segmentation was conducted, so the results are specific to this 2D method. Future research should explore if 3D analyses offer additional predictive benefits. Fifth, interphase alignment was visually assessed instead of using a standardized quantitative procedure. Despite consistent acquisition parameters and protocols, subtle misalignments may still occur. Future research should use a predefined quantitative registration process and assess the reproducibility of features and model performance pre- and post-registration. Additionally, the registration and interpolation methods should be standardized and validated to ensure they don’t alter intensity and texture information before feature extraction. Sixth, a direct comparison between the proposed nomogram and preoperative ultrasound wasn’t done due to the retrospective nature of the study and the lack of standardized ultrasound protocols. Future prospective studies should standardize ultrasound evaluations and directly compare ultrasound, conventional MRI, and DLR-based models. Finally, this retrospective study included only patients who underwent preoperative MRI and thyroidectomy with postoperative pathological confirmation at a single institution. Consequently, selection, referral, and verification biases cannot be excluded.

In conclusion, the proposed nomogram integrating a Min-modality DLR_signature with quantitative MRI parameters showed promising potential for the noninvasive prediction of gross ETE in PTC. These findings support the feasibility of applying MCE MRI post-processing to preoperative ETE assessment. However, given the retrospective single-center design, limited sample size, and absence of a fully independent external test cohort, the diagnostic performance and generalizability of the proposed model remain to be established. Further prospective multicenter studies using a prespecified and locked model are required before clinical implementation.

## Data Availability

The original contributions presented in the study are included in the article/**Supplementary Material**. Further inquiries can be directed to the corresponding author.

## References

[1] Sherman SI . Thyroid carcinoma. Lancet. (2003) 361:501–11. doi: 10.1016/s0140-6736(03)12488-9 12583960

[2] Davies L Roman BR Fukushima M Ito Y Miyauchi A . Patient experience of thyroid cancer active surveillance in Japan. JAMA Otolaryngology-- Head Neck Surg. (2019) 145:363–70. doi: 10.1001/jamaoto.2018.4131 30703198 PMC6481594

[3] Boucai L Zafereo M Cabanillas ME . Thyroid cancer: a review. Jama. (2024) 331:425–35. doi: 10.1001/jama.2023.26348 38319329

[4] Sung H Ferlay J Siegel RL Laversanne M Soerjomataram I Jemal A . Global cancer statistics 2020: GLOBOCAN estimates of incidence and mortality worldwide for 36 cancers in 185 countries. CA: A Cancer J For Clin. (2021) 71:209–49. doi: 10.3322/caac.21660 33538338

[5] Liu L Oh C Heo JH Park HS Lee K Chang JW . Clinical significance of extrathyroidal extension according to primary tumor size in papillary thyroid carcinoma. Eur J Surg Oncol J Eur Soc Surg Oncol Br Assoc Surg Oncol. (2018) 44:1754–9. doi: 10.1016/j.ejso.2018.05.009 29934055

[6] Patti L Gay S Musso L Maltese C Spina B Minuto M . Impact of microscopic extrathyroidal extension on differentiated thyroid cancer post-surgical risk of recurrence: a retrospective analysis. J Endocrinol Invest. (2023) 46:2157–64. doi: 10.1007/s40618-023-02070-y 36976482 PMC10514175

[7] Haugen BR Alexander EK Bible KC Doherty GM Mandel SJ Nikiforov YE . 2015 American Thyroid Association management guidelines for adult patients with thyroid nodules and differentiated thyroid cancer: The American Thyroid Association Guidelines Task Force on Thyroid Nodules and Differentiated Thyroid Cancer. Thyroid: Off J Am Thyroid Assoc. (2016) 26:1–133. doi: 10.1089/thy.2015.0020 26462967 PMC4739132

[8] Amin MB Greene FL Edge SB Compton CC Gershenwald JE Brookland RK . The eighth edition AJCC cancer staging manual: Continuing to build a bridge from a population-based to a more "personalized" approach to cancer staging. CA: A Cancer J For Clin. (2017) 67:93–9. doi: 10.3322/caac.21388 28094848

[9] Shin JH Ha TK Park HK Ahn MS Kim KH Bae KB . Implication of minimal extrathyroidal extension as a prognostic factor in papillary thyroid carcinoma. Int J Surg. (2013) 11:944–7. doi: 10.1016/j.ijsu.2013.06.015 23820062

[10] Hay ID Johnson TR Thompson GB Sebo TJ Reinalda MS . Minimal extrathyroid extension in papillary thyroid carcinoma does not result in increased rates of either cause-specific mortality or postoperative tumor recurrence. Surgery. (2016) 159:11–9. doi: 10.1016/j.surg.2015.05.046 26514317

[11] Tran B Roshan D Abraham E Wang L Garibotto N Wykes J . An analysis of the American Joint Committee on Cancer 8th edition T staging system for papillary thyroid carcinoma. J Clin Endocrinol Metab. (2018) 103:2199–206. doi: 10.1210/jc.2017-02551 29672723

[12] Harries V McGill M Yuan A Wang LY Tuttle RM Shaha AR . Does macroscopic extrathyroidal extension to the strap muscles alone affect survival in papillary thyroid carcinoma? Surgery. (2022) 171:1341–7. doi: 10.1016/j.surg.2021.08.041 34600743 PMC8960478

[13] Chung SR Baek JH Choi YJ Sung TY Song DE Kim TY . Sonographic assessment of the extent of extrathyroidal extension in thyroid cancer. Korean J Radiol. (2020) 21:1187–95. doi: 10.3348/kjr.2019.0983 32729261 PMC7458864

[14] Lamartina L Bidault S Hadoux J Guerlain J Girard E Breuskin I . Can preoperative ultrasound predict extrathyroidal extension of differentiated thyroid cancer? Eur J Endocrinol / Eur Fed Endocrine Societies. (2021) 185:13–22. doi: 10.1530/eje-21-0091 33886499

[15] Qi Q Huang X Zhang Y Cai S Liu Z Qiu T . Ultrasound image-based deep learning to assist in diagnosing gross extrathyroidal extension thyroid cancer: a retrospective multicenter study. EClinicalMedicine. (2023) 58:101905. doi: 10.1016/j.eclinm.2023.101905 37007735 PMC10050776

[16] Hu S Zhang H Sun Z Ge Y Li J Yu C . Preoperative assessment of extrathyroidal extension of papillary thyroid carcinomas by ultrasound and magnetic resonance imaging: a comparative study. La Radiologia Med. (2020) 125:870–6. doi: 10.1007/s11547-020-01184-0 32249390

[17] Javid M Mirdamadi A Javid M Keivanlou MH Amini-Salehi E Norouzi N . The evolving role of MRI in the detection of extrathyroidal extension of papillary thyroid carcinoma: a systematic review and meta-analysis. BMC Cancer. (2024) 24:1531. doi: 10.1186/s12885-024-13288-1 39696088 PMC11657418

[18] Zhong H Wang T Hou M Liu X Tian Y Cao S . Deep learning radiomics nomogram based on enhanced CT to predict the response of metastatic lymph nodes to neoadjuvant chemotherapy in locally advanced gastric cancer. Ann Surg Oncol. (2024) 31:421–32. doi: 10.1245/s10434-023-14424-0 37925653

[19] Gao Y Wang W Yang Y Xu Z Lin Y Lang T . An integrated model incorporating deep learning, hand-crafted radiomics and clinical and US features to diagnose central lymph node metastasis in patients with papillary thyroid cancer. BMC Cancer. (2024) 24:69. doi: 10.1186/s12885-024-11838-1 38216936 PMC10787418

[20] Lin CY Guo SM Lien JJ Lin WT Liu YS Lai CH . Combined model integrating deep learning, radiomics, and clinical data to classify lung nodules at chest CT. La Radiologia Med. (2024) 129:56–69. doi: 10.1007/s11547-023-01730-6 37971691 PMC10808169

[21] Sehgal CM Cary TW Arger PH Wood AK . Delta-projection imaging on contrast-enhanced ultrasound to quantify tumor microvasculature and perfusion. Acad Radiol. (2009) 16:71–8. doi: 10.1016/j.acra.2008.07.013 19064214 PMC2644424

[22] Zhao K Chen C Zhang Y Huang Z Zhao Y Yue Q . Preoperative assessment of Ki-67 labeling index in pituitary adenomas using delta-radiomics based on dynamic contrast-enhanced MRI. J Magnetic Resonance Imaging: JMRI. (2025) 62:508–18. doi: 10.1002/jmri.29764 40091561

[23] Wang W Jin F Song L Yang J Ye Y Liu J . Prediction of peripheral lymph node metastasis (LNM) in thyroid cancer using delta radiomics derived from enhanced CT combined with multiple machine learning algorithms. Eur J Med Res. (2025) 30:164. doi: 10.1186/s40001-025-02438-1 40075509 PMC11905534

[24] Agha RA Mathew G Rashid R Kerwan A Al-Jabir A Sohrabi C . Revised strengthening the reporting of cohort, cross-sectional and case-control studies in surgery (STROCSS) guideline: an update for the age of artificial intelligence. Premier J Sci. (2025) 10:100081. doi: 10.70389/pjs.100081

[25] Liu Y Xiang L Liu FY Yahya N Chai JN Hamid HA . Accuracy of radiomics in the identification of extrathyroidal extension and BRAF(V600E) mutations in papillary thyroid carcinoma: a systematic review and meta-analysis. Acad Radiol. (2025) 32:1385–97. doi: 10.1016/j.acra.2024.11.014 39765435

[26] Yu P Wu X Li J Mao N Zhang H Zheng G . Extrathyroidal extension prediction of papillary thyroid cancer with computed tomography based radiomics nomogram: a multicenter study. Front Endocrinol. (2022) 13:874396. doi: 10.3389/fendo.2022.874396 35721715 PMC9198261

[27] Xu XQ Zhou Y Su GY Tao XW Ge YQ Si Y . Iodine maps from dual-energy CT to predict extrathyroidal extension and recurrence in papillary thyroid cancer based on a radiomics approach. AJNR Am J Neuroradiology. (2022) 43:748–55. doi: 10.3174/ajnr.a7484 35422420 PMC9089265

[28] Wei R Wang H Wang L Hu W Sun X Dai Z . Radiomics based on multiparametric MRI for extrathyroidal extension feature prediction in papillary thyroid cancer. BMC Med Imaging. (2021) 21:20. doi: 10.1186/s12880-021-00553-z 33563233 PMC7871407

[29] Khalil A Rahimi A Luthfi A Azizan MM Satapathy SC Hasikin K . Brain tumour temporal monitoring of interval change using digital image subtraction technique. Front Public Health. (2021) 9:752509. doi: 10.3389/fpubh.2021.752509 34621723 PMC8490781

[30] Jian ZH Li JY Wu KH Li Y Li SX Chen HD . Surgical effects of resecting skull base tumors using pre-operative multimodal image fusion technology: a retrospective study. Front Neurol. (2022) 13:895638. doi: 10.3389/fneur.2022.895638 35645981 PMC9133916

[31] Kim S Mannelli L Hajdu CH Babb JS Clark TW Hecht EM . Hepatocellular carcinoma: assessment of response to transarterial chemoembolization with image subtraction. J Magnetic Resonance Imaging: JMRI. (2010) 31:348–55. doi: 10.1002/jmri.22038 20099348

[32] Ni Z Zhou T Fang H Lin X Xing Z Li X . Radiomics and deep learning for large volume lymph node metastasis in papillary thyroid carcinoma. Gland Surg. (2024) 13:1639–49. doi: 10.21037/gs-24-308 39421056 PMC11480870

[33] Hao Y Pan C Chen W Li T Zhu W Qi J . Differentiation between Malignant and benign thyroid nodules and stratification of papillary thyroid cancer with aggressive histological features: whole-lesion diffusion-weighted imaging histogram analysis. J Magnetic Resonance Imaging: JMRI. (2016) 44:1546–55. doi: 10.1002/jmri.25290 27093648

[34] Hu S Zhang H Wang X Sun Z Ge Y Li J . Can diffusion-weighted MR imaging be used as a tool to predict extrathyroidal extension in papillary thyroid carcinoma? Acad Radiol. (2021) 28:467–74. doi: 10.1016/j.acra.2020.03.005 32303443

