# Supplementary material for: Prediction of gross extrathyroidal extension in papillary thyroid carcinoma using a deep learning radiomics model based on post-processing techniques for multi-phase contrast-enhanced MRI images
Source: Front Endocrinol (Lausanne). 2026 Sep 29;17:1917885. doi: 10.3389/fendo.2026.1917885 (PMC13657937; doi:10.3389/fendo.2026.1917885)
Supplement: Supplementary file 1 [file SupplementaryFile1.docx]

**Supplementary Material**

**Table E1.** Parameters of MRI sequence

| **MR scanner** | **MRI sequence** | **Plane** | **Repetition time**  **(ms)** | **Echo time**  **(ms)** | **Slice thickness**  **(mm)** | **Slice gap**  **(mm)** | **Number of excitations** | **Field of view**  **(cm)** | **Matrix size** | **Sequence** |
| --- | --- | --- | --- | --- | --- | --- | --- | --- | --- | --- |
| 1.5 Tesla | T2WI with fat suppression | Coronal | 1300 | 81.2 | 4 | 0.5 | 4 | 20 | 288×192 | FRFSE |
|  | T1WI | Axial | 540 | 10.6 | 4 | 0.5 | 2 | 20 | 288×224 | FSE-XL |
|  | T2WI with fat suppression | Axial | 3000 | 86.9 | 4 | 0.5 | 2 | 20 | 320×224 | FRFSE |
|  | DWI | Axial | 6550 | 85 | 4 | 0.5 | 8 | 20 | 128×128 | SE-EPI |
|  | Contrast-enhanced T1WI | Axial | 5.5 | 1.6 | 4 | -2 | 1 | 25 | 256×192 | FSPGR |

Abbreviations: T2WI, T2-weighted image; T1WI, T1-weighted image; DWI, diffusion-weighted imaging; FRFSE, fast recovery fast spin echo; FSE, fast spin echo; SE-EPI, spin echo echo-planar imaging; FSPGR, fast spoiled gradient echo.

**Table E2** The diagnostic performance of radiomics model using optimal machine learning algorithms based on six imaging modalities for predicting gross ETE

| **Models** | **Cohort** | **Accuracy** | **AUC (95% CI)** | | **Sensitivity** | **Specificity** | **PPV** | **NPV** |
| --- | --- | --- | --- | --- | --- | --- | --- | --- |
| MCE_P7 | Training | 0.754 | 0.81 | 0.7069 - 0.9131 | 0.68 | 0.795 | 0.654 | 0.814 |
| LR | Validation | 0.7 | 0.679 | 0.4636 - 0.8953 | 0.636 | 0.737 | 0.583 | 0.778 |
| MCE_P2 | Training | 0.824 | 0.905 | 0.8351 - 0.9755 | 0.875 | 0.795 | 0.7 | 0.921 |
| LR | Validation | 0.774 | 0.798 | 0.6320 - 0.9645 | 0.583 | 0.895 | 0.778 | 0.773 |
| ADD | Training | 0.9 | 0.92 | 0.8446 - 0.9954 | 0.76 | 0.978 | 0.95 | 0.88 |
| SVM | Validation | 0.806 | 0.772 | 0.5656 - 0.9783 | 0.667 | 0.895 | 0.8 | 0.81 |
| ADD/SUB | Training | 0.843 | 0.891 | 0.8125 - 0.9697 | 0.6 | 0.978 | 0.937 | 0.815 |
| ExtraTrees | Validation | 0.645 | 0.754 | 0.5832 - 0.9256 | 0.917 | 0.474 | 0.524 | 0.9 |
| Max | Training | 0.714 | 0.83 | 0.7365 - 0.9240 | 0.96 | 0.578 | 0.558 | 0.963 |
| NaiveBayes | Validation | 0.613 | 0.623 | 0.4163 - 0.8293 | 0.833 | 0.474 | 0.5 | 0.818 |
| Min | Training | 0.829 | 0.87 | 0.7806 - 0.9598 | 0.72 | 0.889 | 0.783 | 0.851 |
| LightGBM | Validation | 0.71 | 0.805 | 0.6473 - 0.9623 | 0.5 | 0.842 | 0.667 | 0.727 |

**Abbreviations:** ETE, Extrathyroidal Extension; PPV, Positive Predictive Value; NPV, Negative Predictive Value; AUC, Area Under the Curve; LR, Logistic Regression; SVM, Support Vector Machines; ExtraTrees, Extremely Randomized Trees; LightGBM, Light Gradient Boosting Machine.

**Table E3** The diagnostic performance of the DTL model for predicting gross ETE based on six image processing modalities

| **Models** | **Cohort** | **Accuracy** | **AUC (95% CI)** | | **Sensitivity** | **Specificity** | **PPV** | **NPV** |
| --- | --- | --- | --- | --- | --- | --- | --- | --- |
| MCE_P7 | Training | 0.714 | 0.747 | 0.6234-0.8709 | 0.600 | 0.778 | 0.600 | 0.778 |
|  | Validation | 0.700 | 0.586 | 0.3457-0.8265 | 0.364 | 0.895 | 0.667 | 0.708 |
| MCE_P2 | Training | 0.871 | 0.931 | 0.8646-0.9967 | 0.880 | 0.867 | 0.786 | 0.929 |
|  | Validation | 0.677 | 0.632 | 0.4184-0.8448 | 0.500 | 0.789 | 0.600 | 0.714 |
| ADD | Training | 0.714 | 0.751 | 0.6315-0.8707 | 0.560 | 0.800 | 0.609 | 0.766 |
|  | Validation | 0.581 | 0.700 | 0.5095-0.8896 | 0.833 | 0.421 | 0.476 | 0.800 |
| ADD/SUB | Training | 0.843 | 0.906 | 0.8385-0.9739 | 0.680 | 0.933 | 0.850 | 0.840 |
|  | Validation | 0.710 | 0.660 | 0.4474-0.8728 | 0.417 | 0.895 | 0.714 | 0.708 |
| Max | Training | 0.729 | 0.809 | 0.7071-0.9107 | 0.760 | 0.711 | 0.594 | 0.842 |
|  | Validation | 0.710 | 0.697 | 0.4898-0.9050 | 0.500 | 0.842 | 0.667 | 0.727 |
| Min | Training | 0.771 | 0.852 | 0.7547-0.9501 | 0.760 | 0.778 | 0.655 | 0.854 |
|  | Validation | 0.839 | 0.853 | 0.6927-1.0000 | 0.667 | 0.947 | 0.889 | 0.818 |

**Abbreviations:** DTL, deep transfer learning; ETE, extrathyroidal extension; PPV, positive predictive value; NPV, negative predictive value; AUC, area under the curve; MCE, multiphase contrast enhanced.

**Table E4.** Predictive Features Identified by Feature Selection of Deep Learning Radiomics Model based on Late Fusion methods

| **Parameter** | **Features** | **Feature Types** | **Coefficients** |
| --- | --- | --- | --- |
| Min | DTL_Min_signature | Deep Learning | 0.272485 |
|  | wavelet_HLH_glrlm_ShortRunHighGrayLevelEmphasis | GLRLM | 0.139562 |
|  | wavelet_LLH_firstorder_Skewness | firstorder | 0.034213 |
|  | wavelet_LHL_glrlm_LongRunEmphasis | GLRLM | -0.007029 |
|  | wavelet_LHH_glrlm_LongRunLowGrayLevelEmphasis | GLRLM | -0.009188 |
|  | wavelet_LHH_gldm_LowGrayLevelEmphasis | GLDM | -0.030085 |
|  | wavelet_LLH_glrlm_ShortRunHighGrayLevelEmphasis | GLRLM | -0.037797 |
|  | original_glcm_Imc2 | GLCM | -0.039662 |
|  | wavelet_HHL_glrlm_GrayLevelNonUniformityNormalized | GLRLM | -0.047744 |
|  | wavelet_LLL_firstorder_RootMeanSquared | firstorder | -0.058686 |
|  | wavelet_HLH_firstorder_Range | firstorder | -0.065865 |
|  | wavelet_HHL_glrlm_LowGrayLevelRunEmphasis | GLRLM | -0.073722 |
|  | wavelet_LHL_gldm_DependenceNonUniformityNormalized | GLDM | -0.074146 |

**Abbreviations:** DTL, deep transfer learning; GLCM, gray-level co-occurrence matrix; GLRLM, gray-level run-length matrix; GLDM, gray-level dependence matrix.

**Table E5.** Definitions of Terms Used in the Manuscript

| **Standardized term** | **Definition** |
| --- | --- |
| DTL | Deep transfer learning framework based on a pretrained ResNet-101 model was used to extract deep transfer learning features from six derived image modalities and construct the gross ETE prediction models |
| DLR | Deep learning radiomics framework used to extract deep transfer learning features and radiomic features and to construct the gross ETE prediction models |
| DTL_signature | Output probabilities of the gross ETE prediction models constructed using the DTL model for the six derived image modalities |
| DLR_signature | Output probabilities of the gross ETE prediction models constructed using the DLR model for the six derived image modalities |
| DTL_Min_signature | Output probabilities of the gross ETE prediction models constructed using the Min-modality DTL model |
| DLR_Min_signature | Output probabilities of the gross ETE prediction models constructed using the Min-modality DLR model |

**Figure E1.** Diagram of protrusion value. The protrusion value is defined as the length of the perpendicular line extending from the outermost point of the tumor to the line connecting the intersection points of the tumor contour and the thyroid contour.

**Figure E2.** The image processing pipeline for five distinct image modalities. (**a**) The ADD modality image is produced through the fusion of multi-phase contrast-enhanced P2 and P7 images utilizing the ADD method, which involves the summation of pixel values at corresponding locations in the two images. (**b**) The SUB modality image is derived from the fusion of P2 and P7 images using the SUB method, characterized by the subtraction of pixel values at corresponding locations. (**c**) The Max modality image is created by fusing P2 and P7 images through the Max method, which retains the higher pixel value while discarding the lower one at corresponding locations. (**d**) The Min modality image is generated by the fusion of P2 and P7 images using the Min method, which retains the lower pixel value and discards the higher one at corresponding locations. (**e**) The ADD/SUB modality image results from the fusion of the ADD and SUB images using the ADD method.

**Figure E3.** The ResNet101 architecture.

**Figure E4.** Gradient-weighted Class Activation Mapping (Grad-CAM) applied to each image modality

**Figure E5.** Learning curves of the ResNet101 deep learning model in the Min image modality

**Figure E6.** The SHAP (SHapley Additive exPlanations) force plots demonstrate feature contributions to model predictions for two cases. (**a, b**) Left: Min modality images show PTC lesions in the thyroid for Case 1 (**a**, 24-year-old male, right lobe, no gross ETE) and Case 2 (**b**, 31-year-old female, left lobe, with gross ETE). Right: SHAP force plots detail each feature's contribution to the model's prediction. (**c**) The SHAP plot emphasizes the importance of deep learning radiomics features in the LightGBM Min modality model for predicting gross ETE in PTC. Features are ranked by importance, with DTL_Min_signature being the most crucial. The x-axis indicates feature impact: right of 0.0 means increased risk, and left means reduced risk. Feature values are color-coded; higher DTL_Min _signature values imply increased risk, and lower values imply reduced risk.

**Figure E7.** The predictive performance of the nomogram for gross ETE via 10-fold cross-validation in the training and validation cohorts.

**Figure E8.** Receiver operating characteristic curves comparing the conventional MRI parameter model based on protrusion value and ADC_mean, senior radiologist assessment, and the combined nomogram for the preoperative prediction of gross extrathyroidal extension in papillary thyroid carcinoma. The AUCs were 0.852 (95% CI, 0.779–0.925) for the MRI parameter model, 0.664 (95% CI, 0.577–0.750) for senior radiologist assessment, and 0.935 (95% CI, 0.888–0.981) for the nomogram.

**Deep-transfer-learning model optimization**

Deep features were extracted using a ResNet101 initialized with weights pretrained on ImageNet. Transfer learning was adopted instead of training the network from random initialization to reduce the number of parameters that needed to be learned exclusively from the relatively limited study cohort. Before being entered into the network, the images were normalized according to the ImageNet preprocessing scheme. The model was optimized using stochastic gradient descent (SGD), with an initial learning rate of 0.01, a batch size of 32, and a maximum of 70 training epochs. Class-balanced batch sampling was not applied. The pretrained ResNet101 was fine-tuned using the training data, and the validation data were used to monitor the loss during model development. No explicit early-stopping strategy or epoch-wise model-checkpoint selection was specified in the training configuration; therefore, training was conducted for the prespecified number of epochs, and the model obtained at the completion of training was retained for subsequent feature extraction. The training and validation losses were recorded to assess model convergence and to visually examine potential divergence between the two curves. For visualization in Figure E5, the loss curves were smoothed using a Savitzky–Golay filter with a window length of 7; this smoothing was used only for graphical presentation and did not affect model training or parameter optimization.

**Additional Details of Image Preprocessing**

N4 bias-field correction was performed using the SimpleITK framework (version 2.2.1) to mitigate B1-related signal-intensity inhomogeneity. The N4 algorithm was run at four fitting levels, with a maximum of 50 iterations per level and a convergence threshold of 0.001. Following bias-field correction, voxel intensities were linearly normalized to a fixed range of 0–2048 using the same procedure for all patients and image modalities. This normalization did not incorporate pathological labels or outcome information.

All image volumes were subsequently resampled to a standardized voxel size of 0.1 × 0.1 × 1 mm³. Nearest-neighbor interpolation was used for the MRI volumes and segmentation masks. For the masks, this method preserved discrete ROI labels and prevented the generation of fractional boundary values. For the MRI volumes, it was selected to avoid generating intermediate intensity values and interpolation-related smoothing. The same resampling and interpolation settings were applied to all cases and modalities. Although through-plane upsampling may introduce local voxel-replication effects, its potential influence was reduced by the 2D analysis strategy, in which the slice containing the largest tumor area was selected for feature extraction.

The MCE_P2 and MCE_P7 phases were acquired during the same examination using matched spatial geometry. Because the ADD, ADD/SUB, Max, and Min modalities were generated through voxel-wise operations, two radiologists reviewed the paired source phases before image derivation, with particular attention to tumor contours, thyroid boundaries, adjacent anatomical landmarks, interphase displacement, and motion-related ghosting. The source phases and derived images were also reviewed together after image generation. No visually significant spatial mismatch or ghosting artifact was identified in the images included in model development; therefore, no additional deformable registration was performed. This assessment represented visual spatial quality control rather than quantitative registration validation.

The four derived modalities were generated using prespecified voxel-wise mathematical operations in a standardized scripted pipeline. Once the paired source images and preprocessing parameters were fixed, the derivation procedure was deterministic and involved no manual adjustment. The same ROI mask was propagated to all spatially corresponding modalities to ensure anatomical consistency across input channels.

**WSI Processing and Spatial feature extraction**

The tissue cells in whole slide images (WSI) were analyzed using a multi-step approach. The initial step in the processing pipeline involved nuclei segmentation, which was performed using the HoVer-NeXt model, as proposed by Baumann et al. (2024). HoVer-NeXt is a deep learning-based pipeline specifically designed for the fast and accurate segmentation of nuclei in histopathological slides. Precise segmentation of individual nuclei was achieved by the model, which is a crucial step for further cell classification and spatial analysis. After nuclei segmentation, the resulting masks were used to extract the coordinates of each individual nucleus in the image, serving as the foundation for cell classification and subsequent clustering analyses. Following segmentation, cell classification was performed based on their morphological features.The classification was accomplished using the Lizard_convnextv2_large model, which has been fine-tuned for cell classification in pathology images. This model categorizes the segmented cells into seven different types: mitosis, connective, eosinophil, plasma, lymphocyte, neutrophil, and epithelial cells. Each nucleus was assigned a label corresponding to its cell type based on the model's predictions, providing a detailed map of cell distribution across the tissue. This step allowed for a high-resolution characterization of the tissue, identifying cellular composition and distinguishing between the different cell types present in the microenvironment.

Once the cell types were classified, the spatial relationships between the cells were analyzed using DBSCAN (Density-Based Spatial Clustering of Applications with Noise), a density-based clustering algorithm. The primary objective of this clustering step was to identify the spatial organization of cells, which often reveals patterns of cellular interactions within the tissue. The distance threshold for clustering was selected empirically, ensuring meaningful aggregation of cells while maintaining resolution for the finer spatial details. With the clusters identified, we then extracted a variety of spatial features that describe the relationships between the different cell types. For each identified cluster, we calculated the centroid, which served as a representative point of the cluster’s location. The distance between these centroids was then measured to determine the spatial separation between different cell types. Furthermore, the size of each cluster was computed to understand the density and distribution of cells within the tissue. The spatial relationship between clusters was analyzed to investigate how different cell types interact with one another。

In addition to the DBSCAN clustering, a Graph Neural Network (GNN) was constructed based on the spatial coordinates and cell type labels. Each cell was represented as a node in the graph, and edges were established between nodes that were spatially close to one another, with the distance between them acting as a weight for the edge. This approach allowed us to model the tissue as a dynamic network of interacting cells. The network construction process allowed for the extraction of both local features—such as the density of specific cell types within a given neighborhood—and global features, which describe the overall structure of cellular interactions across the entire tissue. From the trained GNN, a set of spatially relevant features were extracted, including node-level features such as the cell type distribution within each neighborhood and graph-level features that capture the overall connectivity patterns across the tissue.

**The detailed definitions of the features are described as follows:**

1.Degree Mean (Degree_Mean)

Definition: The average degree of nodes in the graph, representing the number of edges connected to each node.

Interpretation: Reflects the average number of connections for each cell. A high degree value indicates that cells have more connections, potentially related to interactions or clustering among cells.

2.Degree Standard Deviation (Degree_StdDev)

Definition: The standard deviation of node degrees.

Interpretation: Measures the variance in the number of connections among nodes. A high standard deviation suggests significant differences in the number of connections between cells.

3.Clustering Coefficient Mean (Clustering_Mean)

Definition: The average clustering coefficient of nodes, which represents the proportion of a node's neighbors that are also connected to each other.

Interpretation: A high clustering coefficient indicates that neighboring cells are closely connected, potentially forming small cellular clusters.

4.Clustering Coefficient Standard Deviation (Clustering_StdDev)

Definition: The standard deviation of clustering coefficients.

Interpretation: Measures the variance in the clustering of neighboring cells. A high standard deviation implies that some cells have tightly connected neighbors, while others have fewer connections among their neighbors.

5.Betweenness Centrality Mean (Betweenness_Mean)

Definition: The average betweenness centrality of nodes, indicating how often a node appears on the shortest paths between other nodes.

Interpretation: High betweenness centrality means the cell plays an important role in the cell network, potentially serving as a key hub.

6.Betweenness Centrality Standard Deviation (Betweenness_StdDev)

Definition: The standard deviation of betweenness centrality.

Interpretation: Measures the variance in betweenness centrality. A high standard deviation suggests that some cells are central to the network, while others are less important.

7.Closeness Centrality Mean (Closeness_Mean)

Definition: The average closeness centrality of nodes, representing the average distance from a node to all other nodes.

Interpretation: High closeness centrality indicates that the cell can quickly reach other cells in the network, possibly suggesting its influence within the network.

8.Closeness Centrality Standard Deviation (Closeness_StdDev)

Definition: The standard deviation of closeness centrality.

Interpretation: Measures the variance in closeness centrality. A high standard deviation suggests that some cells are closer to others in the network, while others are more distant.

9.Eigenvector Centrality Mean (Eigenvector_Mean)

Definition: The average eigenvector centrality of nodes, representing a node's influence based on the importance of its neighbors.

Interpretation: High eigenvector centrality indicates that the cell is connected not only to many cells but also to influential ones, implying a higher degree of influence in the network.

10.Eigenvector Centrality Standard Deviation (Eigenvector_StdDev)

Definition: The standard deviation of eigenvector centrality.

Interpretation: Measures the variance in eigenvector centrality. A high standard deviation suggests that some cells have much higher influence than others in the network.

11.Density (Density)

Definition: The density of the graph

Interpretation: Represents the compactness of the cell network. Higher density indicates more connections between cells, suggesting a more tightly connected network.

12.Average Shortest Path Length (Avg_Shortest_Path_Length)

Definition: The average shortest path length between all pairs of nodes in the graph.

Interpretation: Reflects the connectivity of the cell network. Shorter average path lengths indicate tighter connectivity between cells, making the network more efficient.

13.Number of Connected Components (Num_Connected_Components)

Definition: The number of connected components in the graph, i.e., the number of isolated subgraphs.

Interpretation: Indicates the level of network fragmentation. A higher number of components suggests that there are multiple isolated cell clusters in the network.

14.Connection Frequency (Connection_Freq_{type1}_{type2})

Definition: The frequency of connections between cell types type1 and type2.

Interpretation: Displays the frequency of interactions between different cell types, helping to analyze intercellular interactions.

15.Proportion of Each Cell Type (Proportion_{cell_type})

Definition: The proportion of each cell type within the total cell population.

Interpretation: Reflects the distribution of cell types in the overall cell population, helping to understand the composition of the cell community.

16.Average Distance Between Different Cell Types (Avg_Distance_{type1}_{type2})

Definition: The average distance between cell types type1 and type2.

Interpretation: Displays the spatial distribution of different cell types. A larger average distance suggests that the cell types are more dispersed in the tissue.

17.Cell Type Entropy (Cell_Type_Entropy)

Definition: The entropy of the distribution of cell types, measuring the uniformity of the distribution.

Interpretation: High entropy indicates a more uniform distribution of cell types, while low entropy suggests that certain cell types dominate the network.

18.Density of Cell Types (Density_{cell_type})

Definition: The density of each cell type within the total area of the image.

Interpretation: Reflects the spatial density of each cell type. High density indicates clustering of that cell type in specific regions.

19.Moran’s I (Moran_I)

Definition: A spatial autocorrelation index, measuring the degree of clustering of cells of the same type.

Interpretation: A high Moran's I value indicates significant clustering of cells of the same type, while a low value suggests a more uniform distribution.

20.Moran’s I p-value (Moran_p_Value)

Definition: The p-value for the significance test of Moran’s I.

Interpretation: Indicates whether the Moran's I value is significantly different from zero (random distribution). A low p-value suggests significant spatial clustering of cell types.

21.Geary’s C (Geary_C)

Definition: Another spatial autocorrelation index, measuring the similarity between adjacent cell types.

Interpretation: A low Geary's C value indicates high similarity between adjacent cells, suggesting spatial aggregation.

22.Geary’s C p-value (Geary_p_Value)

Definition: The p-value for the significance test of Geary's C.

Interpretation: Indicates whether the Geary’s C value is significantly different from zero (random distribution). A low p-value suggests significant similarity between neighboring cell types.

23.High-High Clusters (High_High)

Definition: The number of clusters with high values.

Interpretation: Reflects the extent of clustering in regions with high values, indicating significant spatial aggregation of certain cell types.

24.Low-Low Clusters (Low_Low)

Definition: The number of clusters with low values.

Interpretation: Reflects the extent of clustering in regions with low values, suggesting significant spatial dispersion of certain cell types.

25.High-Low Clusters (High_Low)

Definition: The number of clusters formed by high and low values.

Interpretation: Indicates the spatial relationship between regions of high and low values, helping to understand spatial distribution patterns of different cell types.

26.Low-High Clusters (Low_High)

Definition: The number of clusters formed by low and high values.

Interpretation: Indicates the spatial relationship between low and high value regions, aiding in the understanding of spatial distribution patterns among cell types.
